# Patient Centeredness in Electronic Communication: Evaluation of Patient-to-Health Care Team Secure Messaging

**DOI:** 10.2196/jmir.8801

**Published:** 2018-03-08

**Authors:** Timothy P Hogan, Tana M Luger, Julie E Volkman, Mary Rocheleau, Nora Mueller, Anna M Barker, Kim M Nazi, Thomas K Houston, Barbara G Bokhour

**Affiliations:** ^1^ Center for Evaluating Patient-Centered Care in the Veterans Health Administration Edith Nourse Rogers Memorial Veterans Hospital Bedford, MA United States; ^2^ Center for Healthcare Organization and Implementation Research Edith Nourse Rogers Memorial Veterans Hospital Bedford, MA United States; ^3^ Division of Health Informatics and Implementation Science Department of Quantitative Health Sciences University of Massachusetts Medical School Worcester, MA United States; ^4^ Psychology Field Group Pitzer College Claremont, CA United States; ^5^ Department of Communication Bryant University Smithfield, RI United States; ^6^ Veterans and Consumers Health Informatics Office Office of Connected Care Veterans Health Administration, US Department of Veterans Affairs Washington, DC United States; ^7^ Department of Health, Law, Policy & Management School of Public Health Boston University Boston, MA United States

**Keywords:** health communication, electronic mail, patient portals, patient-centered care, veterans

## Abstract

**Background:**

As information and communication technology is becoming more widely implemented across health care organizations, patient-provider email or asynchronous electronic secure messaging has the potential to support patient-centered communication. Within the medical home model of the Veterans Health Administration (VA), secure messaging is envisioned as a means to enhance access and strengthen the relationships between veterans and their health care team members. However, despite previous studies that have examined the content of electronic messages exchanged between patients and health care providers, less research has focused on the socioemotional aspects of the communication enacted through those messages.

**Objective:**

Recognizing the potential of secure messaging to facilitate the goals of patient-centered care, the objectives of this analysis were to not only understand why patients and health care team members exchange secure messages but also to examine the socioemotional tone engendered in these messages.

**Methods:**

We conducted a cross-sectional coding evaluation of a corpus of secure messages exchanged between patients and health care team members over 6 months at 8 VA facilities. We identified patients whose medical records showed secure messaging threads containing at least 2 messages and compiled a random sample of these threads. Drawing on previous literature regarding the analysis of asynchronous, patient-provider electronic communication, we developed a coding scheme comprising a series of a priori patient and health care team member codes. Three team members tested the scheme on a subset of the messages and then independently coded the sample of messaging threads.

**Results:**

Of the 711 messages coded from the 384 messaging threads, 52.5% (373/711) were sent by patients and 47.5% (338/711) by health care team members. Patient and health care team member messages included logistical content (82.6%, 308/373 vs 89.1%, 301/338), were neutral in tone (70.2%, 262/373 vs 82.0%, 277/338), and respectful in nature (25.7%, 96/373 vs 33.4%, 113/338). Secure messages from health care team members sometimes appeared hurried (25.4%, 86/338) but also displayed friendliness or warmth (18.9%, 64/338) and reassurance or encouragement (18.6%, 63/338). Most patient messages involved either providing or seeking information; however, the majority of health care team member messages involved information provision in response to patient questions.

**Conclusions:**

This evaluation is an important step toward understanding the content and socioemotional tone that is part of the secure messaging exchanges between patients and health care team members. Our findings were encouraging; however, there are opportunities for improvement. As health care organizations seek to supplement traditional encounters with virtual care, they must reexamine their use of secure messaging, including the patient centeredness of the communication, and the potential for more proactive use by health care team members.

## Introduction

### Background

#### Patient-Centered Care and Communication

The term “patient centeredness,” although still being investigated and refined [1-3], has come to encapsulate the intersection of many priorities and strategies that focus on the unique, individual needs of patients [3]. For example, patient centeredness may simultaneously refer to a broader biopsychosocial perspective on health and illness, a focus on individual patients and the influence that health care provider characteristics or behavior and healing environments can have on the care experience, the sharing of power and responsibility across stakeholders, and the building of therapeutic alliances between patients and providers [4,5]. Perhaps because of the broadness of the concept, health care systems still struggle to translate patient-centered care into practice [1-3,6]. Within the Veterans Health Administration (VA), the Office of Patient-Centered Care and Cultural Transformation (OPCC&CT) has been tasked with leading the system redesign and cultural shift required to provide veterans with care that is more patient centered. Launched in 2012, OPCC&CT defines patient-centered care as care that is “personalized” (tailored to personal goals, history, and lifestyle), “proactive” (preventive care, which leverages holistic approaches), and “patient driven” (led by what matters most to the individual patient) [7,8]. Thus, in VA, patient-centered care is built upon the veteran’s experience, such as healing environments of care and genuine, personal relationships with providers, as well as a focus on personalized care across multiple domains of wellness (ie, mind, body, and spirit). Patient-centered communication is an essential component of this care, aiming to strengthen the patient-provider partnership by eliciting and understanding the patients’ perspectives, needs, and values; providing patients with the information needed to participate in care to the extent that they desire; and building a shared understanding of a health problem and its treatment [9,10]. In VA, OPCC&CT views patient-provider communication as an essential component in understanding the veteran perspective and in fostering true partnerships between veterans and their providers [8].

A substantial body of research employing a variety of methods [11-16] indicates that patient-centered communication affects various health care processes, patient behaviors, and health outcomes [17]. Patient-centered communication improves processes such as increased patient participation during an encounter [13] and patients’ recall of treatment information [18-20]. Influences on more intermediate outcomes are also seen, such as increased satisfaction with care [13,16,21], confidence in communication [22], and improved treatment adherence and appointment follow-up [23]. Moreover, patient-centered communication has been linked to health outcomes such as improved metabolic control and fewer physical limitations in diabetes, better hypertension control, short-term pain control in cancer patients, improved functional status in ulcer patients, less inflammatory organ damage in patients with lupus, and improved emotional well-being [11,17,24]. However, most of this work has focused on the intermittent in-person encounter and has not extended to continuous care supported by technology.

#### Role of Technology in Patient-Centered Communication

Information and communication technologies have the potential to support patient-centered communication by providing patients with health information to prepare for face-to-face visits and engagement in care [25-27] and strengthening the patient-provider relationship [28,29]. Patient-provider email or asynchronous electronic secure messaging enables patients to interact with their health care providers to exchange nonurgent health information [27,29-31] and has been associated with improved chronic disease self-management [32,33], reduced outpatient visits [34], and urgent care utilization [35]. VA has strategically promoted the use of secure messaging toward the goals of improved communication between veterans and their care teams and increased continuity of care [36]. Nevertheless, the adoption of secure messaging and other asynchronous forms of communication may have inherent disadvantages. Nonverbal modes of communication, which often assist to convey context and tone, are significantly limited by the use of electronic communication such as secure messaging [37,38]. As a result, electronic communication may create more psychological distance between parties, reducing the likelihood of secure messaging being effectively used for interpersonal communication goals such as relationship-building [37-39]. The asynchronous nature of secure messaging may also prevent recipients from receiving immediate feedback or clarity about any interpersonal misunderstandings, which can lead to further miscommunication [37]. Yet, others suggest that users have already adapted their communication styles to adjust to electronic mediums by relying on emoticons or emojis and textual emphasis (eg, ALL CAPS) to project relational content [37,38]. Similarly, patients have expressed value in the ability to formulate and articulate their questions for providers at their own convenience [28,30], often feeling more comfortable with disclosing personal details due to the psychological distance mentioned earlier [39,40]. Secure messaging has also been found to lower the threshold at which patients initiate communication, resulting in more interactions between physical encounters and perception of greater access [31].

Although previous research has examined the content of electronic communication between patients and providers extensively [30,41-46], less research has focused on the “socioemotional” aspects of the communication enacted by those messages, including how socioemotional tone is expressed and whether it reflects the patient-centered goals of eliciting patients’ perspectives, addressing their needs, expressing and responding to emotions, and contributing to a therapeutic partnership [28]. One study of secure messaging identified patient frustration with a perceived lack of empathy in some physician-sent electronic communications [30], suggesting that the socioemotional communication that could facilitate patient-centered goals may be lacking in electronic messages. However, given that the ultimate goal of patient-centered care is to be responsive to patient’s needs, what constitutes patient-centered electronic communication may look different for each patient and may need to be tailored to patient-communication styles and preferences. Thus, to further enhance the potential benefits of secure messaging, it is important to explore the various ways that patient centeredness might be realized (or not) through communication via this electronic medium. This is especially important as health care organizations seek to supplement traditional encounters with virtual care.

### Research Questions

The VA is a large, geographically diverse, and integrated care system that has a tethered personal health record (PHR) patient portal. VA patients who use secure messaging are also diverse, and penetration of secure messaging into the veteran population is higher than in the general population. As of December 2017, over 2.5 million veteran patients had access to secure messaging, representing a penetration rate of approximately 42% of the 5.9 million VA patients receiving health care services in fiscal year 2017 [47]. Within this context, we conducted a cross-sectional coding evaluation of a corpus of secure messages exchanged between VA patients and health care team members at different VA facilities. Our work contributes to the limited knowledge of how socioemotional tone can be electronically communicated in the secure messages exchanged between patients and health care team members, along with the content and purpose of those messages. Our research questions included (1) why do patients and health care team members exchange secure messages? and (2) what socioemotional tone do these messages convey?

In the Discussion section, we explore how these aspects of the secure messages exchanged between patients and health care team members might facilitate the goals of patient-centered care.

## Methods

### Study Design and Setting

We identified 8 VA facilities to sample secure messages. Each of these facilities is located in a metropolitan area of the United States; however, because of their catchment areas, they also serve the needs of veterans living in nearby rural areas. We selected these facilities because they are comparable in terms of the diverse patients that they serve, many with complex health care needs, and the wide range of clinical services that they offer.

### Patient-Centered Care and Secure Messaging in the Veterans Health Administration

As part of a system-wide transformational initiative [48], VA’s Primary Care Program Office has implemented a patient-centered medical home model known as Patient Aligned Care Teams (PACT) [49-51]. The principles of the PACT model call for care that is patient-driven, team-based, efficient, comprehensive, continuous, and encompasses good communication and coordination [52]. The use of information and communication technologies is often considered a critical component of patient-centered medical homes [53]. Within the PACT model, asynchronous secure messaging through the VA’s PHR portal, My Health*e*Vet, is envisioned as a means to enhance access to care, support bidirectional communication between patients and health care team members, and supplement other communication mediums [31,54].

In keeping with the PACT model of team-based care, VA implemented secure messaging with a flexible triage team model. Similar to telephone triage, an identified member(s) of the health care team is responsible for reading incoming secure messages and can respond directly or assign action to another member of the triage team, or to another triage team as needed. Health care team members can elect to save all or parts of a secure message or message thread as a progress note in the VA electronic health record (EHR), based on clinical relevance.

### Data Collection: Message Corpus

To select secure messaging threads for our evaluation, we used VA administrative datasets to identify all patients at the 8 facilities whose EHR progress notes showed secure messaging threads containing at least 2 messages (an original message and corresponding responses) between January and July 2013. From this sample of patients, we then gathered a random sample of threads, deidentified them, and copied them into a structured template for analysis. In an effort to represent a variety of patient and health care team profiles, our goal was to assemble a sample comprising 50 secure messaging threads from each of the 8 facilities. For the purposes of this evaluation, the secure messaging threads randomly selected for analysis were gathered between September and November 2013. Key demographic characteristics of the patients represented by the sample of secure messaging threads were obtained from VA administrative datasets. The evaluation was exempt from institutional review board review as part of a larger VA quality improvement initiative.

**Table 1:** Domains for coding secure messages.

Domain	Stakeholder	Codes (subcodes)
Content: The “what” of the message, representing biomedical, holistic, or logistical aspects	Patient and health care team member	Logistical, biomedical, holistic, nonmedical
Socioemotional tone: The “how” of the message, representing the feeling(s) that the message conveys	Patient and health care team member	Attitude (neutral, positive, negative); formality (formal, informal); respectfulness; concern or worry; assertiveness; hurried or rushed; friendliness or warmth; reassurance or encouragement; sympathetic or empathetic; anger or irritation; reflective or legitimizes; depression or sadness
Purpose: The “why” of the message, representing the reason(s) for the message	Patient	Information seeking (proactiveness, treatment or care plan, prescription refill, symptom related, health care team member opinion, test related, referral request, request to fill out form); information provision (health update, responding to health care team member questions); confirmation (gratitude, acknowledgement)
	Health care team member	Information provision (responding to patient questions with pertinent information; giving instructions; providing orientation to medical procedures, therapy, or prevention, checking understanding); information seeking (eliciting patient response regarding treatment or action plan, symptom related, previous treatment plans)

### Coding Scheme of Patient-Centered Communication Elements

Drawing on previous literature regarding the analysis of asynchronous, patient-provider electronic communication [28,30,41,42], we developed a series of a priori patient and health care team member codes to apply to the individual messages appearing in each thread. Following our literature review, conversations within our team, and consultations with other patient-provider communication experts, we organized these codes into 3 domains: (1) message content, (2) message socioemotional tone, and (3) message purpose (Table 1). The purpose and tone domains were further categorized to reflect work by Roter and colleagues [4,28], demonstrating that information exchanged between patients and providers carries emotional meaning, cognitive meaning, affective talk, and instrumental behaviors.

### Coding Reliability

To assess reliability, 3 team members with expertise in qualitative analysis coded an initial subset of 4 secure messaging threads including 8 individual secure messages. The initial inter-rater reliability (N=3) revealed Cohen kappa values from .80 to 1.00 across all codes [55]. The team members then met to discuss discrepancies and revise the coding scheme with examples to clarify conceptual distinctions and to test the enhanced coding schema with an additional 4 secure messages. The full sample of 384 secure messaging threads was then divided among the 3 team members to code independently using QSR International’s NVivo V10 software to support data analysis. The team met weekly to discuss and resolve coding questions. Revisions were made and examples added to the coding scheme as needed. Recognizing that an email message can serve multiple communication functions (eg, information exchange as well as sharing worry or concern) [28], each message in a secure messaging thread could be assigned multiple codes as appropriate, and those codes were not mutually exclusive. In other words, each secure message could be assigned multiple instances of content, socioemotional tone, or purpose. The exceptions to this practice were the codes “formal” and “informal,” which were assigned once to each message. Due to the complexity of the coding scheme, intercoder reliability was assessed at weekly meetings before discussing coding questions. The 3 coders maintained their coding reliability of .80 to 1.00 across all codes (average Cohen kappa=.88) throughout the analysis. In the results below, coding frequencies are summarized at the individual message level and the totals can equal more than 100%. Although this does mean that an individual message represents multiple instances of content and socioemotional tone, it follows rigorous coding strategies typical of such analyses [56]. In keeping with contemporary qualitative analysis, our goal was to provide a snapshot of the content and socioemotional tone represented in these secure messages rather than a definitive measurement of prevalence.

## Results

### Characteristics of Patients and Health Care Team Members

The secure messages in our sample were sent by 292 unique patients and 205 unique VA health care team members across the 8 facilities. As indicated in Table 2, the majority of the patients were male, white, and not of high economic need, meaning that their income was above the threshold set by VA to be eligible for cost-free health care. Their mean age was 59.6 years. VA utilizes a system called the Rural-Urban-Commuting Areas to distinguish between urban and rural areas in the United States based on patient zip codes of residence [57]. Although 86% of the patients sending messages were from urban areas, nearly 14% of the patients were from rural areas. The health care team members responding to the messages were largely registered nurses or physicians; fewer messages were sent by nursing assistants or other team members.

**Table 2:** Patient and health care team member characteristics.

Patient characteristics^a^		Value
Age (mean, SD)		59.6 (12.3)
**Gender, n (%)**		
	Male	242 (85.5)
	Female	41 (14.5)
**Race, n (%)**		
	White	207 (73.1)
	African-American	31 (11.0)
	Unknown or missing	39 (13.8)
	Other	6 (2.1)
**Socioeconomic status, n (%)**		
	High economic need	62 (21.9)
**Geographic location, n (%)**		
	Urban	245 (86.6)
	Rural	38 (13.4)
Elixhauser comorbidity index (mean, SD)		2.9 (2.3)
**Role of health care team member, n (%)**		
	Registered nurse	84 (41.0)
	Physician	65 (31.7)
	Nursing assistant	30 (14.6)
	Other	10 (4.9)
	Advanced practice nurse	9 (4.4)
	Physician assistant	3 (1.5)
	Psychologist	2 (1.0)
	Medical assistant	1 (<1.0)
	Social worker	1 (<1.0)

^a^Data missing for 9 patients.

### Message Characteristics

Across the 8 facilities, there were differences in the number of secure messaging threads that health care team members had elected to save into the EHR as a progress note over the selected 6-month evaluation period. As such, we were able to gather an average of 48 secure messaging threads from each facility (min=37, max=51) for a total sample of 384 threads comprising 711 individual secure messages. Of the 384 secure messaging threads, most were initiated by patients (90.9%, 349/384) rather than a health care team member (9.1%, 35/384). Of the 711 individual messages, roughly half were sent by patients (52.5%, 373/711), and half were sent by health care team members (47.5%, 338/711). Finally, most patient messages appeared to be composed by the patient him/herself (92.2%, 344/373) as opposed to a proxy (eg, a family member or other informal caregiver; 7.8%, 29/373).

### Message Content

Table 3 presents our content codes with exemplary quotes from patient and health care team member messages.

#### Messages Sent by Patients

The majority of patient secure messages included logistical content (82.6%, 308/373) such as scheduling an appointment or requesting a prescription refill. Half of the patient messages also included biomedical content (50.4%, 188/373), such as mentioning specific diseases, medications, or treatments. Almost 10% of the patient messages (8.6%, 32/373) included holistic content, discussing psychosocial aspects of health, such as exercise, stress management, or family relationships. Fewer messages contained nonmedical content (6.4%, 24/373) such as mention of a change of email address.

#### Messages Sent by Health Care Team Members

The majority of health care team member secure messages were largely logistical in nature (89.1%, 301/338), and many contained biomedical content (29.6%, 100/338). Less than 5% of the messages sent by health care team members were coded as containing holistic content (4.4%, 15/338) but 8.0% contained nonmedical content (27/338), such as acknowledging a holiday or an occasion in one’s personal life.

**Table 3:** Examples of patient and health care team member message content.

Code and stakeholder		Sample message excerpt	Code presence, n (%)
**Logistical content**			
	Patient	“I will be at [location] on Tuesday, March 26 and would like to go to the Dental Clinic to begin some long overdue dental work”	308 (82.6)
	Health care team member	“Wheelchair referral has been placed. Dental consult only good for 72 hours - so I can place the consult closer to the time you would like to go to the clinic”	301 (89.1)
**Biomedical content**			
	Patient	“When you’re back on duty, please be good enough to enter a refill for me for Lisinopril 40MG. My BP lately ranges from 120’s to low 150’s over 60’s / 70’s”	188 (50.4)
	Health care team member	“Upon review of your records both x-rays and medications; your x-rays show that you have early degenerative changes to both knees (arthritis)”	100 (29.6)
**Nonmedical content**			
	Patient	“Here is that link I promised for the ‘Battlefield Of The Mind’ documentary. Fortunately, I am not in it but I contributed money to its making. If you like it after watching, Please buy a DVD copy at the second website. It’s only a few dollars.”	24 (6.4)
	Health care team member	“Hi [name], Happy Mother's Day to you as well! I hope you had a great one.”	27 (8.0)
**Holistic health content**			
	Patient	“I don't know what else to do. I don't want to quit work without knowing that financially I can't support my family. I don't want to cause any more stress on myself although work in itself is stressful”	32 (8.6)
	Health care team member	“I have entered a consult for the Move program. This is a weight management program for veterans”	15 (4.4)

### Message Socioemotional Tone

Tables 4 and 5 presents our socioemotional tone codes with exemplary quotes from patient and health care team member messages.

#### Messages Sent by Patients

Patient messages were frequently coded as neutral in tone (70.2%, 262/373), being direct, to-the-point, and transactional. The remainder of the patient messages were equally positive (14.2%, 53/373) or negative (14.2%, 53/373) in tone. Positive messages reflected patient optimism toward health conditions or treatment plans. Negative messages reflected pessimism stemming from perceptions of health conditions, treatment plans, or actual treatments received. In terms of emotions, almost half of the messages expressed some concern or worry (39.9%, 149/373). Patient messages also exhibited respectfulness (25.7%, 96/373), being mannerly and considerate of the potential feelings and situations of health care team members. However, messages were also equally assertive (25.5%, 95/373) or direct. Many messages conveyed friendliness or warmth (14.5%, 54/373), reading as chatty or chummy and attempting to engage health care team members. Fewer messages demonstrated being reassured or encouraged (1.9%, 7/373), where patients expressed relief and an optimistic outlook about their health or treatments. Patient messages were also coded as more informal (61.1%, 228/373), lacking proper grammar or a salutation and signature, rather than formal (38.9%, 145/373).

#### Messages Sent by Health Care Team Members

Similar to patient messages, the tone of health care team member messages was largely neutral (82.0%, 277/338) rather than positive (14.2%, 48/338) or negative (2.7%, 9/338), and exhibited respectfulness (33.4%, 113/338). At times, the messages appeared hurried or rushed (25.4%, 86/338), although a substantial portion displayed friendliness or warmth (18.9%, 64/338) and offered reassurance or encouragement to patients (18.6%, 63/338). More than half of the secure messages sent by health care team members were coded as informal (59.2%, 200/338) rather than formal (40.8%, 138/338).

### Message Purpose

Tables 6 and 7 present our purpose codes with exemplary quotes from patient and health care team member messages.

#### Messages Sent by Patients

Most patient messages involved either providing or seeking information. In terms of information provision, just under half of the messages were coded as a health update (48.8%, 182/373) in which the patient informed a health care team member about some aspect of their current health and well-being.

Regarding information seeking, patient messages requested information regarding treatment or care plans (22.5%, 84/373), prescription refills (22.0%, 82/373), symptoms (16.1%, 60/373), or test results (13.7%, 51/373). Almost a quarter of the messages were coded as proactive in nature (23.9%, 89/373), where patients took initiative to ask questions, express disagreement, or actively contribute to the management of their care. Patients also sought the opinions of health care team members regarding various care-related issues (15.5%, 58/373). Distinct from providing and seeking information, a smaller number of patients sent confirmatory messages to acknowledge receipt of a health care team member message (1.6%, 6/373) or to express gratitude to them (7.0%, 26/373).

#### Messages Sent by Health Care Team Members

The majority of the secure messages sent by health care team members responded to patient questions with information of some kind (72.8%, 246/338). Health care team members also used secure messaging for giving instructions or providing patients with specific action steps toward care (30.5%, 103/338). Over a quarter of the messages also offered orientation to procedures, therapies, or prevention behaviors (26.3%, 89/338). Few of the health care team member messages reflected information seeking; only 5.6% (19/338) were coded as eliciting a patient response regarding plans for treatment or a future course of action. In addition, few health care team members utilized secure messaging as a way to ask patients about symptoms (3.3%, 11/338) or previous treatment plans (3.0%, 10/338).

**Table 4:** Examples of patient message tone.

Code	Sample message excerpt	Code presence, n (%)
Neutral attitude	“Just wanted you to know that the MRI is scheduled for June 4, 2013 at 10:30 AM”	262 (70.2)
Positive attitude	“Good news! I'm up and getting around some with the ortho boot and walker. In fact, yesterday and today I actually made it outside over a high doorsill and one step on my own! 3 times today!”	53 (14.2)
Negative attitude	“We tried that already and it didn't work. I realize that things have to be shown not to work before they are changed, but in the mean time I am still gagging and getting headaches”	53 (14.2)
Informal	“I need another holder for the eye drops as the rubber seam still splits around the bottle. Can you pls reorder?”	228 (61.1)
Formal	“[Dr. name], I request a refill of my monthly supply of: (RX# [prescription number]) ACETAMIN 325MG/OXYCODONE 5MG TAB, dispensed on 5 April 13. I am available to pick up medication at the [location] Clinic Pharmacy on 3 May 13 due to the 5th of May is a Sunday. Thank you, [patient name]”	145 (38.9)
Concern or worry (includes anxiety or nervousness)	“[Dr. name], I have been anxiously waiting on your call since this morning. I called the Heart Clinic and the Echocardiogram has been read, dictated and is in the system. PLEASE call me with these results. I've been sick with worry.”	149 (39.9)
Respectfulness	“[Dr. name], I am flying out of town, for work, Monday at 1:00pm. If possible, I would like pick my monthly Methadone prescription at the Pharmacy window, Monday morning at 9:00am? As always, thank you for your help. Respectfully, [Patient name]”	96 (25.7)
Assertiveness	“I need you to put in my order for the lab to take blood. I thought [Dr.name] had done it, but there’s no order.”	95 (25.5)
Friendliness or warmth	“Good afternoon [Dr. name], hope all is well. I am requesting to have the following medications renewed: Diclofenac and Pravastatin. Also, I received my card for my 6-month follow-up, so if possible, I would like to set up that appointment at your earliest convenience. Thank you, have a good day!”	54 (14.5)
Hurried or rushed	“checking on status of morphine rx.. also need Dilantin, zomig, and ceterzine refilled, thx.”	33 (8.8)
Anger or irritation	“I do not need gauze sponges and it seems every time I try to get drain sponges I get gauze sponges. I at least need to have the order for drain sponges available so I can go to pharmacy and pick them up to avoid further problems. These items are very similar and this happens all the time because they are so similar. But I need DRAIN SPONGES.”	26 (7.0)
Depression or sadness	“My Dad passed away on the 9th and I am having a hard time. I feel so empty and lost. I miss him so much.”	9 (2.4)
Reassurance or encouragement	“The extra dose of Lopressor seems to be working. Thanks for the new BP machine.”	7 (1.9)
Sympathetic or empathetic	—	—
Reflective or legitimizes	—	—

**Table 5:** Examples of health care team member message tone.

Code	Sample message excerpt	Code presence, n (%)
Neutral attitude	“Your medication has been refilled”	277 (82.0)
Positive attitude	“I’m glad that your range of motion is improving, even if only slightly to begin with”	48 (14.2)
Negative attitude	“I referred you to rehab. I have no quick answer for your pain. With your chronic osteoarthritis of the knees, knee pain will always be there. The goal is to bring the pain level down so you can function better but to get rid of it totally, this may not be a realistic goal”	9 (2.7)
Informal	“done, and given to pharmacy”	200 (59.2)
Formal	“[Patient name], I hope you are well. I see that you did not make your appointment to the endocrinologist. I believe we need to get their opinion as well and then I would like to see you again. Most sincerely, [Dr. name]”	138 (40.8)
Respectfulness	“Good morning, I will renew both the pseudoephedrine and saline for mail. Have a great week, [Dr. name]”	113 (33.4)
Hurried or rushed	“I have written the scripts and will be sent to VA pharmacy today.”	86 (25.4)
Friendliness or warmth	“I will have our clerk get you scheduled. They may be in the process. That is a week of vacation for me that accidentally wasn't blocked earlier this year. Sorry about rescheduling. We will send you a new appt. Hope your wife has a full, speedy recovery.”	64 (18.9)
Reassurance or encouragement	“Feel free to message me whenever you need to – you are not bugging me!”	63 (18.6)
Assertiveness	“Your Lantus Rx has no more refills and has to be renewed. You should have enough to cover you till close to end of March according to your chart. [Dr name] will be made aware in order to renew and have it mailed to you. Thank you.”	41 (12.1)
Sympathetic or empathetic	“I am so sorry to hear you have not been feeling well. I will give you a call to discuss.”	28 (8.3)
Reflective or legitimizes	“Hi [son’s name], I do think it is reasonable to consider rivastigmine, but the VA does not yet have the transdermal patch. We do have the pill formulation which I believe has a slightly higher rate of side effects.”	17 (5.0)
Concern or worry (includes anxiety or nervousness)	“I am sorry to hear that you fell- please come to the ER if it happens again. Did you end up going to a local hospital?”	15 (4.4)
Anger or irritation	“I am not exactly sure why u are emailing me every day about your nutritional data. I do not know who asked you to do this. I know that I have not.”	3 (0.9)
Depression or sadness	—	—

**Table 6:** Examples of patient message purpose.

Code and subcode		Sample message excerpt	Code presence, n (%)
**Information-seeking**			
	Proactiveness	“[Dr name], I have used up all of the Clotrimazole you prescribed for me. I still have the itching on the middle portion of my body. Is there something else that will work better, a spray or something like that?”	89 (23.9)
	Treatment or care plan	“Hello, [Dr. name], When I was at your office yesterday my blood pressure was high. I checked it today and it’s still running high: 147/95. Maybe it’s time for a new blood pressure medication. I have gotten older since you prescribed Lisinopril 5mg.”	84 (22.5)
	Prescription refill	“I also need a prescription for my nitroglycerine tablets. My current supply is about to expire.”	82 (22.0)
	Symptom related	“I would like to make an appointment to check on a swelling that is taking place below and to the right of my tongue. No pain or sensations, just an obvious swelling beneath the outside skin.”	60 (16.1)
	Health care team member opinion	“They also want me to change the Meloxicam for Tramadol, Naproxen and time-scheduled Tylenol. This was after I wrote and requested the 90 prescription of Meloxicam. I would truly like to hear your opinion on this.”	58 (15.5)
	Test related	“By any chance, have my HIV results come back yet?”	51 (13.7)
	Referral request	“I was wondering if you can put in referrals for me for Neurology and Endocrinology. I need to see someone about the migraines and also about my pituitary growth.”	22 (5.9)
	Request to fill out form	“My job gave me the form for disability and there is a portion for you to complete”	18 (4.8)
**Information provision**			
	Health update	“Another interesting factoid: yesterday, I weighed myself. I got out of the shower and I weighed 170 pounds! So that means, since May, I lost about 20 to 25 pounds.”	182 (48.8)
	Responding to health care team member questions	“It was many years ago, maybe 3 or 4. Don't remember the dosage.”	9 (2.4)
**Confirmation**			
	Gratitude	“Both my wife and I really want to thank you for your patience and care last week. Although you may feel that you were just doing your job, to us, it meant so much.”	26 (7.0)
	Acknowledgment	“Thank you. I will make this work.”	6 (1.6)

**Table 7:** Examples of health care team member message purpose.

Code and subcode		Sample message excerpt	Code presence, n (%)
**Information provision**			
	Responding to patient questions with pertinent information	“Just got it back. It is normal. You should recheck in 4 months.”	246 (72.8)
	Giving instructions	“Please stop by 6C to give a urine sample and also to have [name] or any Medical Assistant check your blood pressure and record - it was a bit high on recent check.”	103 (30.5)
	Providing orientation to medical procedures, therapy, or prevention	“Although the pulses in your feet are fine, we can send you to the [location] VA for Ankle Brachial Indices testing. This tests your blood pressure in your upper extremities relative to your lower extremities (down to your toes). This is a first-line test in evaluating the circulation in your legs.”	89 (26.3)
	Checking understanding	“I thought we were going to do it through the hematology clinic given your previous events? Adding [Dr. name] for opinion.”	20 (5.9)
**Information seeking**			
	Eliciting patient response regarding treatment or action plan	“You have arthritis in the knees - would you like a referral for exercise therapy?”	19 (5.6)
	Symptom related	“Are you having any vision problems now?”	11 (3.3)
	Previous treatment plans	“Januvia, or sitagliptin, is a restricted drug. I can place a nonformulary request if you like. If so, I need to know what diabetes meds you have tried that did not work out.”	10 (3.0)

## Discussion

### Principal Findings

We analyzed a sample of secure messages between VA patients and health care team members to understand why these messages are exchanged, and what socioemotional tone the messages convey. Overall, our findings regarding message content are consistent with prior studies. However, our findings related to message tone and implications for emotional expression extend the existing literature.

#### Message Content

We examined message content to place our findings about the tone and purpose of the messages in our sample into a broader context. Our analysis revealed that the majority of patient secure messages included logistical content, demonstrating the organizing efforts of patients to ensure that they had the resources (eg, prescriptions) needed for their own care. These findings are consistent with previous studies, including some studies conducted in VA, highlighting the frequent use of email and secure messaging by patients to address administrative issues and related care actions [29,41,42,44-46]. Although there may be a tendency to view such content as uncomplicated or routine, we believe that it underscores the importance of secure messaging as a tool that patients use to promote care coordination. Patients must often play an active role in coordination [58]; the prevalence of logistical content in our message sample indicates that electronic communication is commonly used by patients to facilitate at least some of this work.

Half of the patient messages also included specific biomedical content, most often the formal names of health conditions and prescription medications, and considerably less holistic and nonmedical content. Health care team member messages were similar, not surprisingly, given that most were responses to secure messages initiated by patients. This point raises important questions about the intended uses of secure messaging, an issue we examine below.

#### Message Socioemotional Tone

Few prior studies have evaluated message tone, a notable exception being Roter et al [28], who found that in a convenience sample of email exchanges between 8 doctor-patient dyads, patients often used email to convey their emotional state, frequently an expression of worry or concern. Our analysis extends beyond Roter et al and found that both the patient and the health care team member secure messages were largely neutral in tone and also tended to exhibit an informal style. This is not to say, however, that messages were devoid of emotion. On the contrary, many messages initiated by patients expressed concern and worry, warmth, or even anger. An important lesson from our analysis is that the neutral tone and informal style characterizing some patient messages should not distract health care providers from the emotions that patients may convey through this medium.

Several positive tonal elements were prominent in the health care team member messages in our sample, including being respectful of patients, showing friendliness or warmth toward them, and offering them reassurance or encouragement. These findings again map to Roter et al [28], who found that many physicians expressed concern, reassurance, partnership, and other supportive socioemotional expressions in response to patient messages containing emotional content. Socioemotional tone can function in interpersonal communication to build rapport and strengthen the relationship between parties [28,59], contributing to the therapeutic partnership that is central to patient-centered care. Still, one-fourth of the health care team member messages in our sample were coded as hurried or rushed. Taken together, these findings suggest that there is considerable variation in the tone of secure messages sent by VA health care team members. It is important to frame our findings about the tone of health care team member messages in the context of the workflow that surrounds secure messaging in VA. Per VA policy, team members are expected to respond to patient messages within 3 business days, and triaging approaches are often used to assign messages to appropriate team members to ensure efficient responses. These realities, coupled with large patient panels and tightly scheduled clinics, may explain the number of messages coded as informal, hurried, or rushed, and those that appeared less sympathetic, reflective, or legitimizing of patient concerns.

#### Message Purpose

Nearly half of the patient messages in our sample were coded as information updates, in which they informed health care team members about some aspect of their health. Patient provision of such updates has been documented in previous studies [42,44,46] and is a powerful illustration of how secure messaging can facilitate the shift from episodic to continuous care and cultivate ongoing, healing relationships as argued for by the Institute of Medicine [60]. In addition to providing updates, patients also used secure messaging to seek information about a variety of topics. Although some of these topics, such as prescription refills and test results, reflect the high prevalence of logistical content in our messages, other topics, such as symptoms and team member opinions, suggest that patients also use secure messaging to seek information about more nuanced topics.

Our exploration of message purpose did reveal gaps. The numerous instances of information provision coded in health care team member messages is not surprising—the majority involved responding to patient questions with information, and over a quarter gave instructions or offered orientation to some health-related topic. Eliciting the patient’s perspective has been described as an important element of patient-centered communication [9]; however, there were few instances in our data where health care team members appeared to use secure messaging to reach out to patients and seek information from them regarding treatment plans. Even in their replies, it was uncommon for health care team members to ask for patient input on topics such as treatment plans or descriptions of symptoms. These trends highlight the reactive nature that tends to characterize much secure messaging use among health care team members, similar to in-person encounters.

### Practice Implications

Our analysis offers a snapshot of the electronic communication between the patients and health care team members represented by the secure messages in our sample. Although the literature has suggested that being responsive to patient emotions and concerns can build rapport and contribute to a therapeutic relationship [28,59], we must caution that our evaluation is not intended to serve as a determination of whether the secure-messages in our sample were in fact “patient-centered.” As discussed above, we identified considerable variation in tone among health care team member messages. However, placing our findings in the context of patient-centered communication may suggest ways that the content and socioemotional tone of these secure messages could facilitate the goals of patient-centered care. For example, we coded ample instances of health care team members being respectful, sympathetic, friendly, reflective, and reassuring, which could indicate a response to patient-expressed emotion. Yet, we also coded many instances of health care team members seeming hurried or rushed in their messages. Although at face value, messages coded as the former could be considered “more patient-centered” and messages coded as the latter could be considered “less patient-centered,” we must remember that patient centeredness can encompass a wide range of behaviors. It is likely that some patients would prefer receiving messages from their health care team members that reflect elements of friendliness or sympathy; however, there are likely other patients who would prefer receiving messages that are succinct, timely, and to-the-point; messages that, in our sample, would likely have been coded as hurried or rushed. These points underscore the importance of understanding and embracing patient preferences for communicating with members of their health care team and using technologies such as secure messaging. It may be that true patient centeredness in secure messaging involves health care team members discussing upfront with their patients what exactly they want from their communication in this medium and, in turn, tailoring their approach to that individual patient. Future work should examine ways of eliciting patient preferences for secure messaging, testing different approaches for implementing those preferences into practice, and assessing their impact on patient satisfaction and other outcomes.

Although what might be considered “patient-centered” content and socioemotional tone in secure messaging is likely to vary with patient preferences, we suggest that there are ways in which elements of patient-centered communication can be further integrated into asynchronous electronic communication to promote the therapeutic relationship. These opportunities are critical given the goals of many health care systems to increase secure messaging use and to expand virtual care. The variation in tone of health care team member messages points to the importance of cultivating secure messaging practices that fully elicit the patient’s perspective, and empower the patient to participate in their care to the extent they desire. Doing so requires recognition that patients and health care team members are both active producers of meaning when using secure messaging [61], and that messages are themselves more than simple chunks of information. On the contrary, in addition to their purpose, many of the patient messages in our sample included expressions of socioemotional tone. The fact that patient secure messages could at once be conveying emotions, offering important contextual detail, and attempting to accomplish care-related tasks underscores the complexity that can characterize communication in this medium. A multiple-goals perspective [62] that recognizes that patients and health care team members can have a variety of goals when they send a secure message may be a valuable way to frame future secure messaging studies and to understand its use in practice. Applying such a perspective would enable a richer understanding of the complexity of secure messaging communication while also offering analytical tools (eg, types of goals, types of responses to goals) to support more nuanced analyses.

Similarly, the preponderance of secure messages in our sample in which health care team members responded to a patient request or inquiry, and the limited use of the medium by health care team members to reach out to patients or to seek information from them represents a significant missed opportunity to promote patient participation, engagement, and relationship building. Advocating for more “proactive” uses of secure messaging in which health care team members initiate communication, elicit patients’ perspectives, and draw them into relevant dialogue would constitute a paradigm shift in current approaches to this communication medium. Health care team members and patients alike will need different training about secure messaging if it is envisioned as much as a medium for engaging patients and bolstering the therapeutic relationship as a medium for addressing logistical needs [28,29]. Corresponding workflow implications for the health care team will also have to be examined.

### Limitations

There are several limitations to our evaluation. As noted earlier, the messages that were included in our analysis were only those that the VA health care team members determined clinically relevant to save in the EHR. Additionally, although our goal was to assemble a sample comprising 50 secure messaging threads from each of the participating facilities, in 1 facility, there were fewer than 50 threads in the EHR for the evaluation’s selected time period. Our evaluation also focused solely on secure messaging to the exclusion of other communication mediums. We are unable to ascertain whether other types of communication transpired before, during, or after a secure messaging exchange. As others have similarly argued [46], a different analysis that situates secure messaging in the context of other communication mediums and focuses on how those mediums could augment one another would provide additional insights about the content and socioemotional tone evident in secure messages and how secure messaging is being used to address particular patient needs. Finally, although we report basic information about the veteran patients and health care team members who are represented by the secure messages in our sample, we did not construct the sample to explore associations between the patient and health care team member characteristics and our coding domains.

Acknowledging these limitations, we believe there is value in offering the following best practices to inform health care team members’ use of asynchronous, electronic secure messaging with patients. These best practices extend previous guidelines for electronic communications and the use of email with patients [63,64]:

- Elicit and understand the preferences each patient may have for communicating with their health care team members through secure messaging. As noted above, what constitutes patient centeredness for one patient may be different for another. Health care team members should discuss the use of secure messaging with each of their patients, preferably at the time the patient is adopting the technology, to set expectations and discuss what the patient hopes to ascertain from communication through this medium. In addition to addressing the content and tone that is part of secure message exchanges, such discussions can also foster patient understanding of a health care system’s approach to triaging and processing messages.
- Recognize that expressions of emotion can be an inherent part of patient secure messages. Patients may have various goals in mind when they send a secure message to members of their health care team, and the expression of emotion may be part of those goals. Health care team members should appreciate the presence of emotion as one of the complexities of communicating with their patients through this medium.
- Utilize, as appropriate, patient expressions of emotion as a means to enhance the therapeutic relationship. If the expression of emotion is a natural part of some patient secure messages, not addressing those emotions could be a missed opportunity for health care team members to engage with patients and to use them as a means to foster rapport, shared understandings, and engagement.
- Leverage asynchronous, electronic secure messaging as a means to reach and engage patients. In the current paradigm, much secure messaging use is reactive in nature. A more proactive approach that involves health care team members initiating communication with patients through this medium to seek information and elicit their perspectives could be an effective means of fostering participation in the care process to the extent the patient desires.

### Conclusions

Our evaluation represents an important step toward understanding the content and socioemotional tone that is part of the secure messages exchanged between patients and health care team members, and how asynchronous communication might facilitate the goals of patient-centered care. Our findings suggest that there are opportunities to enhance communication in this medium. The rapid implementation of secure messaging across health care systems places a premium on pursuing such improvements in the short run so that desirable process outcomes and longer term clinical outcomes can be realized through its use.

## Abbreviations

EHR: electronic health record
OPCC&CT: Office of Patient-Centered Care and Cultural Transformation
PACT: Patient Aligned Care Teams
PHR: personal health record
VA: Veterans Health Administration
